# Tolerance and Resistance of *Pseudomonas aeruginosa* Biofilms to Antimicrobial Agents—How *P. aeruginosa* Can Escape Antibiotics

**DOI:** 10.3389/fmicb.2019.00913

**Published:** 2019-05-03

**Authors:** Oana Ciofu, Tim Tolker-Nielsen

**Affiliations:** Department of Immunology and Microbiology, Faculty of Health and Medical Sciences, Costerton Biofilm Center, University of Copenhagen, Copenhagen, Denmark

**Keywords:** *Pseudomonas aeruginosa*, biofilm, antibiotic, tolerance, resistance

## Abstract

*Pseudomonas aeruginosa* is one of the six bacterial pathogens, *Enterococcus faecium*, *Staphylococcus aureus*, *Klebsiella pneumoniae*, *Acinetobacter baumannii*, *Pseudomonas aeruginosa,* and *Enterobacter* spp., which are commonly associated with antimicrobial resistance, and denoted by their acronym ESKAPE. *P. aeruginosa* is also recognized as an important cause of chronic infections due to its ability to form biofilms, where the bacteria are present in aggregates encased in a self-produced extracellular matrix and are difficult or impossible to eradicate with antibiotic treatment. *P. aeruginosa* causes chronic infections in the lungs of patients with cystic fibrosis and chronic obstructive lung disease, as well as chronic urinary tract infections in patients with permanent bladder catheter, and ventilator-associated pneumonia in intubated patients, and is also an important pathogen in chronic wounds. Antibiotic treatment cannot eradicate these biofilm infections due to their intrinsic antibiotic tolerance and the development of mutational antibiotic resistance. The tolerance of biofilms to antibiotics is multifactorial involving physical, physiological, and genetic determinants, whereas the antibiotic resistance of bacteria in biofilms is caused by mutations and driven by the repeated exposure of the bacteria to high levels of antibiotics. In this review, both the antimicrobial tolerance and the development of resistance to antibiotics in *P. aeruginosa* biofilms are discussed. Possible therapeutic approaches based on the understanding of the mechanisms involved in the tolerance and resistances of biofilms to antibiotics are also addressed.

## Introduction

Research done in the last three decades has shown that bacteria in most settings live in the biofilm mode of growth, whereas the planktonic single cell state is considered a transition phase. The shift of bacteria from the planktonic mode of growth to the biofilm state is dependent on the production of adhesins and extracellular matrix components that serve as a scaffold and encase the bacteria in the biofilms (Tolker-Nielsen, 2015). The matrix in *Pseudomonas aeruginosa* biofilms consists mainly of polysaccharides, proteins, extracellular DNA and lipids, and its composition is strain dependent, and also depends on the growth conditions and the age of the biofilm (Pamp et al., 2007). Many *P. aeruginosa* strains are capable of synthesizing the three exopolysaccharides, Pel, Psl, and alginate, which play a role in biofilm formation as matrix components (Høiby et al., 1974; Govan and Deretic, 1996; Friedman and Kolter, 2004; Matsukawa and Greenberg, 2004; Overhage et al., 2005; Ma et al., 2006, 2009). Along with the exopolysaccharides, proteins such as type IV pili, Cup fimbria, CdrA adhesins, LecAB lectins, and Fap amyloid fibers can be part of the *P. aeruginosa* biofilm matrix (O’Toole and Kolter, 1998; Vallet et al., 2001; Klausen et al., 2003; Tielker et al., 2005; Diggle et al., 2006; Giltner et al., 2006; Borlee et al., 2010; Dueholm et al., 2013; Rybtke et al., 2015a). Furthermore, extracellular DNA (eDNA) functions as an important matrix component in *P. aeruginosa* biofilms (Whitchurch et al., 2002; Allesen-Holm et al., 2006). In addition, evidence has been provided that rhamnolipids are involved in the formation of microcolonies in *P. aeruginosa* biofilms (Pamp and Tolker-Nielsen, 2007).

The secondary messenger cyclic diguanosine-5′-monophosphate (c-di-GMP) is a key regulator of the biofilm lifecycle in many bacteria, including *P. aeruginosa* (Fazli et al., 2014; Jenal et al., 2017). High cellular levels of c-di-GMP induce the production of adhesins and extracellular matrix components, which lead to biofilm formation, whereas low c-di-GMP levels downregulate the production of adhesins and extracellular matrix components and cause biofilm dispersal so that the bacteria engage in the planktonic mode of growth. The synthesis and degradation of c-di-GMP in bacteria occur through the opposing activities of diguanylate cyclases (DGCs) and c-di-GMP phosphodiesterases (PDEs). Many of the DGCs and PDEs contain sensory domains that are thought to enable the bacteria to respond to environmental cues and adjust their production of biofilm matrix components. Some of the two component signaling systems in *P. aeruginosa* have been linked to regulation of the production of extracellular matrix components (Goodman et al., 2004; Barraud et al., 2009; An et al., 2010; Malone et al., 2010; Petrova and Sauer, 2010; Moscoso et al., 2011; Li et al., 2013). As a prominent example, the GacA/GacS two component system regulates the expression of a number of genes including those encoding synthesis of the Pel and Psl exopolysaccharides, and it has been shown to intersect with c-di-GMP signaling (Goodman et al., 2004, 2009; Moscoso et al., 2011). Quorum sensing (QS) also affects biofilm formation by *P. aeruginosa* (Davies et al., 1998). The QS system in *P. aeruginosa* consists of the two acyl homoserine lactone-based systems Las and Rhl, and the quinolone-based system PQS, which are interconnected and regulate each other in a complex fashion (Juhas et al., 2005). PQS is positively regulating the production of the eDNA matrix component (Allesen-Holm et al., 2006), whereas the Rhl system regulates rhamnolipid production, which is important for biofilm formation, and the tolerance of *P. aeruginosa* biofilms to immune cells (Pamp and Tolker-Nielsen, 2007; Van Acker et al., 2009).

One of the most important features of microbial biofilms is that the bacteria are able to survive antibiotic treatment administered at high doses (Costerton et al., 1999). If the biofilm is dispersed, the planktonic bacteria show sensitivity to antibiotics and display low minimal inhibitory concentration (MIC) values. The term “tolerance” distinguishes this type of biofilm-associated antibiotic treatment survival from “resistance,” which is characterized by increased MICs and a resistant phenotype of the bacteria dispersed from biofilm. Mechanistically, resistance is due to acquired mutations and usually involves antibiotic-modulating enzymes, efflux pumps, or mutations that eliminate the molecular target of the antibiotic and allows bacteria to survive the antibiotic treatment even if not embedded in a biofilm. In contrast, the antibiotic-tolerant cells in biofilms are able to survive the high antibiotic concentrations only if embedded in the biofilms. Both resistance and tolerance are involved in the recalcitrance of biofilms to antibiotic treatment (Lebeaux et al., 2014).

The term antibiotic tolerance can also be used in the context of planktonic bacterial populations, and here it describes bacterial cells that survive treatment with bactericidal antibiotics without having acquired antibiotic resistance determinants. Antibiotic tolerance of planktonic bacteria is mainly caused by an altered physiological state of the cells as a consequence of environmental stress, and is mediated by cellular stress responses and related systems (Brauner et al., 2016; Trastoy et al., 2018). In biofilms, attached or not to surfaces, bacteria are aggregated in a self-produced extracellular matrix, forming a structured environment, which is not encountered by planktonic cells. The biofilm-specific environment triggers the development of tolerant subpopulations, which constitute a large fraction of the biofilm, and the tolerance mechanisms, for example, related to the extracellular matrix or to the anaerobic conditions (Brauner et al., 2017; Trastoy et al., 2018), which are distinct from those of planktonic cells.

Biofilms are the cause of persistent infections associated with a variety of medical implants, and are also connected with diseases such as chronic wounds, chronic obstructive pulmonary disease, urinary tract infections, and cystic fibrosis (Costerton et al., 1999; Tolker-Nielsen, 2014; Ciofu et al., 2015; Rybtke et al., 2015b). The ability of microbial biofilms to tolerate antibiotics and components of the host immune system is the primary reason for the problematic infections they are causing (Høiby et al., 2015). The currently used antibiotics may decrease the number of bacteria in biofilms, but they cannot completely eradicate the biofilms (Fernandez-Barat et al., 2017), and hence relapses of biofilm infections often occur. Therefore, removal of infected tissues or implanted devices, and subsequent long-term antimicrobial therapy may be required for treatment of biofilm infections, if possible. It has been demonstrated that young biofilms are much more susceptible to antibiotics than more developed biofilms (Hengzhuang et al., 2014; Stewart, 2015), underlining the importance of early interventions in the treatment of biofilm infections. The main clinical consequence of tolerance of biofilms to antibiotics is that the high concentration of antibiotics required for treating biofilm infections [for some antibiotics up to 1,000 times higher than for planktonic cells (Macia et al., 2014)] cannot be achieved *in vivo* by systemic administration without toxicity (Hengzhuang et al., 2012). Providing high antibiotic concentrations through topical administration, combined antimicrobials and sequential therapies or the use of adjuvants to improve the efficacy of antibiotics are therapeutic strategies that are employed or have been proposed to treat biofilm infections (Høiby et al., 2015; Ciofu et al., 2017).

**Figure 1:**
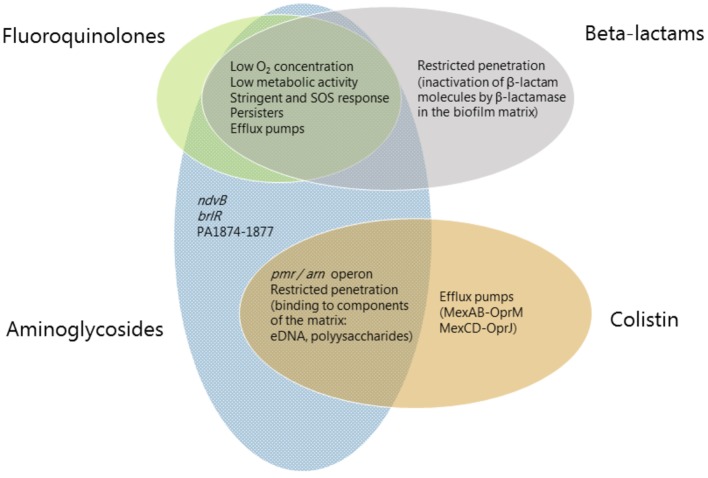
Mechanisms involved in biofilm-associated tolerance against different classes of antibiotics. Beta-lactams are represented in gray ellipses, fluoroquinolones in green, aminoglycosides in blue and antimicrobial peptides (colistin) in orange. Mechanisms which confer tolerance to several classes of antibiotics are listed in the area where the ellipses overlap. Mechanisms which are specific to an antibiotic class are listed in the respective ellipse area.

A detailed understanding of the mechanisms that are involved in the recalcitrance of biofilms toward antimicrobial activity will ultimately enable us to develop efficient treatments against a wide range of persistent infections. Therefore, more research is conducted in order to shed light on the molecular mechanisms that are involved in biofilm-associated antimicrobial tolerance. In the present review, we first describe general mechanisms that contribute to biofilm-associated antimicrobial tolerance. Subsequently, we describe the contribution of specific genes to biofilm-associated antibiotic tolerance. Then, to emphasize the multifactorial nature of biofilm tolerance, we focus on specific classes of antibiotics, and describe biofilm-associated antimicrobial tolerance mechanisms that play a role in tolerance to each of these antibiotic classes (Figure 1). Finally, mechanisms involved in development of mutational antibiotic resistance of biofilm cells are described.

## General Mechanisms of Biofilm Tolerance

The mechanisms of biofilm-associated antibiotic tolerance described here are based on work with *P. aeruginosa*, but may also be relevant for biofilms formed by other organisms (Hall and Mah, 2017).

### Physical Tolerance—Restricted Penetration

When antibiotics are used in the attempt to cure biofilm infections, they must cross the extracellular biofilm matrix in order to reach the embedded bacteria. A number of studies have suggested that biofilm matrices do not inhibit diffusion of antibiotics in general, but that restricted penetration of antibiotics through biofilms may occur in cases, where the antibiotics bind to components of the biofilm matrix or the bacterial membranes (Figure 1; e.g., Walters et al., 2003; Tseng et al., 2013). By the use of fluorescently labeled antibiotics, Tseng et al. (2013) demonstrated directly that the positively charged tobramycin is sequestered at the periphery of *P. aeruginosa* biofilms, whereas the neutral ciprofloxacin readily penetrates into the biofilms. Because the biofilm matrix can be saturated with the antibiotics that it binds, antimicrobial tolerance caused by hindered penetration may be only temporary. However, it might allow the bacteria enough time to adapt to a more tolerant state (Bagge et al., 2004b; Pamp et al., 2008; Kindrachuk et al., 2011). Furthermore, the mechanism may be relevant for infections, where antibiotic concentrations at the site of infection are low so that the antibiotics cannot saturate the biofilm matrix.

Staudinger et al. (2014) reported that when *P. aeruginosa* were grown as aggregates independent of the production of exopolysaccharides in viscous environments that restrain bacterial motility, then wild type bacteria and an exopolysaccharide-deficient *pelApslBCDalgD* mutant strain displayed the same level of antibiotic tolerance. Based on these results, it was suggested that the extracellular matrix does not play a role in the antimicrobial tolerance displayed by *P. aeruginosa* aggregates. However, subsequently, it was demonstrated that biofilm matrix over-expression, as displayed by various clinical isolates, significantly protects *P. aeruginosa* aggregates against antimicrobial treatment (Goltermann and Tolker-Nielsen, 2017). Alginate-overproducing *mucA* mutant bacteria growing in aggregates showed highly increased antibiotic tolerance compared to wild type bacteria in aggregates. Similarly, aggregates formed by *P. aeruginosa wspF* and *yfiR* mutants, which over-produce Pel and Psl exopolysaccharide, showed highly increased antibiotic tolerance compared to wild type bacteria growing in aggregates. The increased antibiotic tolerance was directly attributable to overproduction of the exopolysaccharides, since additional mutations that rendered the *mucA*, *wspF,* and *yfiR* strains deficient in exopolysaccharide synthesis resulted in wild type levels of antibiotic tolerance. Overproduction of biofilm matrix components increased the tolerance of *P. aeruginosa* aggregates toward tobramycin but also to some degree toward ciprofloxacin. As diffusion of ciprofloxacin should not be limited by the matrix components, this may indicate that the presence of the matrix components altered the physiology of the bacteria in the aggregates, for example, by restricting penetration of nutrients or oxygen into the aggregates.

### Physiological Tolerance—Slow Growth and Persisters

**Figure 2:**
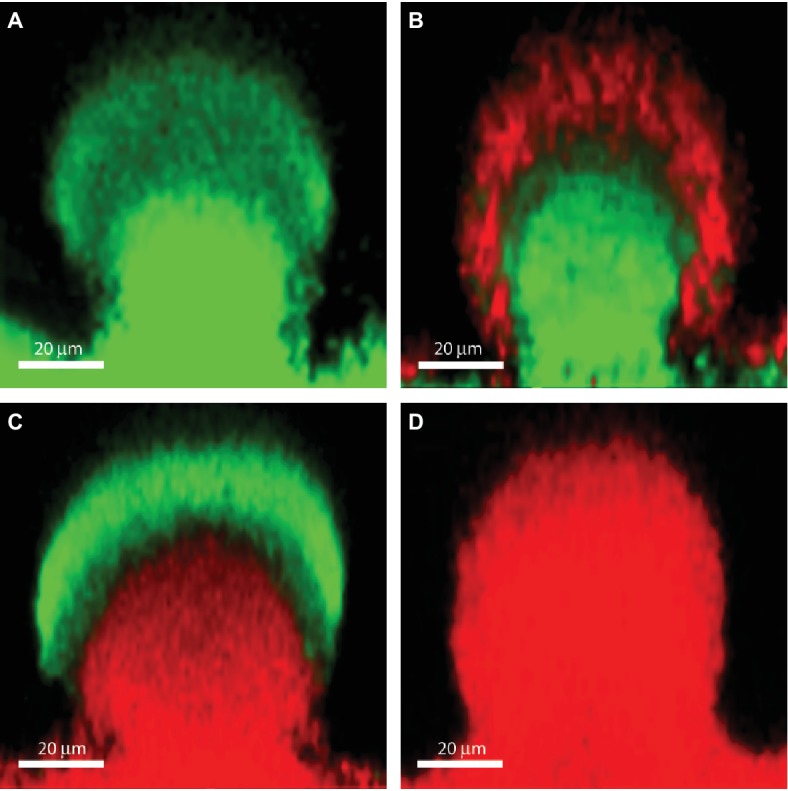
The effect of ciprofloxacin, colistin and the combination of the two antibiotics on *P. aeruginosa* PAO1 biofilm subpopulations. The images show vertical sections through flow-chamber-grown *P. aeruginosa* biofilm microcolonies that were treated with either no antibiotic as control **(A)**, ciprofloxacin **(B)**, colistin **(C)**, or ciprofloxacin and colistin **(D)**. Live bacterial cells appear green due to expression of *gfp*, whereas dead bacterial cells appear red due to staining with propidium iodide (Pamp et al., 2009). Figure is adapted from Cytometry Part A 75:90–103 with permission from the John Wiley and Sons.

Biofilms contain bacterial subpopulations characterized by a wide distribution of metabolic activity. Subpopulations at the periphery of the biofilm display high physiological activity, whereas subpopulations located in the inner parts of the biofilms display low physiological activity or no growth (Pamp et al., 2008; Stewart et al., 2016). This heterogeneity is due to consumption of oxygen and nutrients by the metabolically active bacteria located in the periphery of the biofilm, leaving little or no oxygen and nutrients to the bacteria located in the interior of the biofilm (Stewart et al., 2016). The direct consequence of low-metabolic rates for antibiotic tolerance is the inactivity of the major antibiotic targets, thus affecting in different degrees the efficacy of all bactericidal antibiotics. For example, low-protein synthesis alters the effectiveness of inhibitors of protein-synthesis such as aminoglycosides, low DNA synthesis affects the effect of quinolones and low peptidoglycan production affects the effect of beta-lactams (Figure 1). Figure 2B shows the distribution of live and dead bacteria in a ciprofloxacin-treated *P. aeruginosa* biofilm microcolony grown in a flow chamber.

The poor effect of antibiotics on bacteria with low metabolic activity is in addition to target inactivity also caused by adaptive stress-responses such as the SOS (Bernier et al., 2013) and stringent response (Nguyen et al., 2011). The stringent response is an adaptive response to nutrient and iron starvation, which might occur in biofilms in some environments, and it has been shown to play an important role in tolerance of biofilms to antibiotics (Nguyen et al., 2011). In many bacterial species, including *P. aeruginosa*, starvation activates the stringent response by inducing *relA* and *spoT* gene products to synthesize the alarmone (p)ppGpp, which regulates the expression of many genes. Nguyen et al. (2011) demonstrated an increased susceptibility to fluoroquinolones, meropenem, colistin, and gentamycin of biofilms formed by a *P. aeruginosa* Δ*relAspoT* double knockout mutant, and provided evidence that this phenotype was due to impaired anti-oxidant capacity and consequently increased endogenous oxidative stress. However, in the case of colistin, this is in contradiction to reports showing that the bactericidal effect of this antibiotic is independent of ROS (OH^.^) production (Brochmann et al., 2014; Kolpen et al., 2016). Moreover, stringent response activation was found to prevent accumulation of HAQ pro-oxidants (Nguyen et al., 2011; Schafhauser et al., 2014).

The SOS response may be an important mechanism of bacterial survival under stress conditions. The proteins that are involved in SOS response include the transcriptional repressor LexA and the DNA-binding activating protein RecA (in *E. coli*). In biofilms, the SOS response can be activated due to several biofilm-specific stresses such as oxidative stress (Boles and Singh, 2008) and nutrient starvation, and it has been shown to be induced in an age-dependent manner in *E. coli* biofilms causing tolerance to the fluoroquinolone ofloxacin (Bernier et al., 2013).

Another adaptive starvation response to nutrient limitation was shown to occur through the involvement of the catabolite repressor control protein Crc, which downregulates the metabolic activity of the cells in biofilms. Biofilms formed by a *P. aeruginosa crc* mutant were more susceptible to ciprofloxacin (which affects the metabolically active cells) and more tolerant to colistin (which is effective on metabolically inactive cells) than wild type biofilms (Zhang et al., 2012). Thus, the metabolically inactive subpopulation in *P. aeruginosa* biofilms does not arise solely due to nutrient limitation. It appears that the cells sense limited nutrient conditions and then differentiate into metabolically inactive subpopulations through Crc, possibly as a survival strategy (Zhang et al., 2012). Because oxygen is sparingly soluble and is rapidly respirable by aerobic microorganisms, oxygen concentration gradients are a common feature of bacterial biofilms (Stewart et al., 2016). Thus, oxygen concentrations have been shown by microelectrode profiling to rapidly decrease in *P. aeruginosa* biofilms (Walters et al., 2003). The finding of upregulation of the global, O_2_-sensing, anaerobic regulator Anr in transcriptomic studies of biofilm populations confirmed that cells in biofilms experience hypoxia (Stewart et al., 2015). These anoxic or hypoxic conditions lead to reduced bacterial growth rates (Williamson et al., 2012; Sønderholm et al., 2018). Studies of an *in vitro* colony biofilm model have suggested that 70% (62% ciprofloxacin, 69% tobramycin, and 110% ceftazidime) of the antibiotic tolerance can be explained by oxygen limitation under these conditions (Borriello et al., 2004). *P. aeruginosa* can grow anaerobically by utilizing alternative electron acceptors such as NO_3_^−^ and NO_2_^−^ for respiration through denitrification, or by arginine fermentation. However, these alternative pathways have lower energy efficacy than aerobic respiration (Arai, 2011). In an alginate bead biofilm model, Sønderholm et al. showed that the fastest growth rate was obtained with a combination of O_2_ and NO_3_^−^, enabling both aerobic respiration and denitrification (Sønderholm et al., 2017). The lack of oxygen caused decrease in the metabolic activity of the cells in the deeper layer of the biofilm altering the effectiveness of beta-lactams, fluoroquinolones, and aminoglycosides. An exception is polymyxins and other membrane-targeting compounds such as SDS, EDTA, and chlorhexidine, which preferentially kill the non-growing biofilm bacteria (Pamp et al., 2008; Chiang et al., 2012; Brochmann et al., 2014; Kolpen et al., 2016).

Another tolerance mechanism connected to the low oxygen tension in biofilms is due to the requirement of oxygen molecules for formation of ROS (hydroxyl radicals), which have been shown to be important for the bactericidal effect of antibiotics (Dwyer et al., 2015; Van Acker and Coenye, 2017). Antibiotic-induced ROS production have been described during treatment of *P. aeruginosa* and *Proteus mirabilis* biofilms with quinolones (Aiassa et al., 2010; Jensen et al., 2014) and *Burkholderia cenocepacia* biofilms with aminoglycosides (Van Acker et al., 2013). Biofilms formed by mutants lacking anti-oxidants systems, such as catalases (*katA* for *P. aeruginosa* and *katB* for *B. cenocepacia*) showed increased sensitivity to antibiotics. The anti-oxidant systems, such as catalases and superoxide dismutase, are upregulated by the activation of the stringent response in biofilms (Khakimova et al., 2013; Martins et al., 2018) improving in this way, the antioxidant capacity of the biofilms and thus their tolerance to antibiotics.

As bacterial metabolism plays an important role for the tolerance of biofilms to antibiotics, several strategies targeting energy production have been proposed to overcome the antibiotic tolerance (Meylan et al., 2018).

Antibiotic tolerance can also be caused by the formation of so-called persister cells. Persister cells are slowly-dividing or non-dividing bacteria that are less vulnerable to antibiotics than the bulk of the bacterial population, and when antibiotic treatment is terminated, these cells can transform to a vegetative state and reconstitute infections (Lewis, 2010). The fraction of persister cells in biofilms is usually low (~0.01%), and they should be distinguished from the spatially confined subpopulation of tolerant, metabolically inactive bacteria, which constitute a large fraction of the biofilm, and are inactive because they lack nutrients. Persister cells are believed to be the result of bacterial differentiation into a dormant state. The reduced metabolism exhibited by persister cells evidently enables them to escape the activity of antibiotics that target fundamental cellular processes (e.g., replication, translation, or cell wall synthesis) (Figure 1). Mutant screens have provided evidence that a number of genes (e.g., *rpoS*, *spoT*, *relA*, *dksA*, *dinG*, *spuC*, *algR*, *pilH*, *ycgM*, and *pheA*) are involved in persister formation in *P. aeruginosa*, suggesting that multiple pathways can lead to the development of persister cells (Murakami et al., 2005; Viducic et al., 2006; De Groote et al., 2009). Work with *E. coli* has suggested that the second messenger ppGpp drives persister formation through the Lon protease and activation of toxin-antitoxin (TA) modules (Harms et al., 2016). However, more recent work has shown that the data suggesting the involvement of TA modules in persister cell formation were influenced by the inadvertent infection of mutant strains with bacteriophage ϕ80. Experiments performed with un-infected bacteria no longer support a role of TA modules in *E. coli* persister formation (Harms et al., 2017).

Because persister cells may play an important role in persistent infections, efforts have been done to revive persister cells in order to restore their antibiotic susceptibility. Mannitol was demonstrated to increase the aminoglycoside tobramycin’s efficacy against *P. aeruginosa* biofilm persister cells (Barraud et al., 2013). This effect was blocked by the addition of a proton motive force inhibitor or in a *P. aeruginosa* mutant strain unable to metabolize mannitol, suggesting that mannitol reverts the persister phenotype through an active physiological response of the bacteria. Thus mannitol, which is known to improve CF lung function by facilitating mucus clearance, may also enhance antibiotic sensitivity of CF lung biofilms (Barraud et al., 2013). The different anti-persisters’ strategies have recently been reviewed by Jan Michiels group (Defraine et al., 2018).

## Biofilm Tolerance Caused by the Expression of Specific Genes

A number of genes have been identified as being specifically involved in the tolerance of *P. aeruginosa* biofilms to antibiotics. We use the tolerance term in this case because these genes are expressed specifically in biofilms and therefore mediate biofilm-associated recalcitrance to antibiotics, but do not mediate antibiotic resistance in planktonic cultures. In this section, we describe the involvement of *brlR* and efflux pump genes in antibiotic tolerance, but more examples of specific genes involved in biofilm-associated antibiotic tolerance are given below in the section focusing on tolerance to specific antibiotic classes.

Evidence for a role of the messenger molecule c-di-GMP in antibiotic tolerance of *P. aeruginosa* biofilms was provided by Gupta et al. (2014). As described in the introduction section, the level of c-di-GMP is elevated in biofilm cells in comparison to in planktonic cells. Artificial increase of the c-di-GMP level to “biofilm” levels in planktonic cultures was found to lead to a higher tolerance of the bacterial cells to antibiotics. PA3177 was identified as an active diguanylate cyclase (DGC), whose inactivation rendered biofilm cells sensitive to tobramycin (Poudyal and Sauer, 2018b). It was also shown that PA3177 contributes to biofilm susceptibility in a manner dependent on the levels of c-di-GMP and BrlR. The biofilm resistance locus regulator, BrlR, is a Mer-like transcriptional activator of biofilm-specific antibiotic tolerance in *P. aeruginosa*. Biofilms formed by a *brlR* mutant were found to be more susceptible to various antibiotics than wild-type biofilms, whereas planktonically grown *brlR* mutant bacteria were as susceptible to the antibiotics as the wild type (Liao and Sauer, 2012). Evidence was provided that BrlR is a transcriptional activator of the efflux pumps encoded by the *mexAB-oprM* and *mexEF-oprN* operons, and transcriptional downregulation of these genes was found to be an important factor for the antibiotic susceptibility phenotype observed for *brlR* biofilms (Liao et al., 2013). Furthermore, it was shown that the activity of the BrlR transcriptional regulator is stimulated by binding of c-di-GMP (Chambers et al., 2014). BrlR activates also the expression of ABC transporters, with the ABC transporter PA1874-1877 directly contributing to the antibiotic tolerance of biofilms (Poudyal and Sauer, 2018a). Zhang and Mah (2008) showed that PA1874-1877 was 10 times more highly expressed in *P. aeruginosa* biofilms compared to planktonic cells and that deletion of the genes encoding this pump increases susceptibility of biofilms to tobramycin, gentamycin, and ciprofloxacin while planktonic susceptibility was not affected. Deletion of the genes encoding this pump (PA1874-1877) resulted in an increase in the sensitivity of *P. aeruginosa* biofilms to tobramycin and gentamicin. In addition to affecting the expression of genes encoding proteins involved in efflux pumps and ABC-multidrug transporters, BrlR also affects the expression of genes encoding modification of LPS and membrane protein composition, as well as metabolism and energy generation. This multitude of different potential targets might explain the involvement of BrlR in the regulation of biofilm tolerance to various antibiotics (Poudyal and Sauer, 2018a). However, in contrast to the results described above, Stewart et al. (2015) were unable to detect a difference in biofilm-associated antibiotic tolerance to tobramycin between wild-type *P. aeruginosa* and the *brlR* mutant. It is possible that the difference in tolerance to tobramycin between the wild-type and the *brlR* mutant is observed only at high antibiotic concentrations, which were not investigated by Stewart et al. (2015).

Bacterial multidrug efflux pumps are capable of pumping antibiotics out of the bacterial cell *via* an energy requiring process dependent on membrane potential and ATP. Different forms of stresses can induce efflux pumps, such as induction in *P. aeruginosa* of MexXY-OprM by oxidative stress, MexEF-OprN by nitrosative stress, and MexCD-OprJ by membrane-damaging agents (Poole, 2014). As these types of stresses might be encountered in biofilms, they might lead to induction of efflux pumps in *P. aeruginosa* biofilms contributing to antibiotic tolerance. In other species, such as *Burkholderia cenocepacia*, efflux pumps have been shown to be involved in the tolerance of biofilms to tobramycin and ciprofloxacin (Buroni et al., 2014).

## Tolerance of *in Vivo* Biofilms

Different antibiotic tolerance mechanisms may be of importance for the survival of biofilms in different environments (Hall and Mah, 2017). *In vivo,* the local biofilm environments may be quite variable, depending on the site of infection. For example, pharmacokinetic/pharmacodynamic studies have shown that in order to overcome the antibiotic tolerance of biofilms in mouse lungs, high doses and/or prolonged treatment are required (Hengzhuang et al., 2012). These high dosages cannot be administered systemically to the patients in the clinical settings due to toxicity but can be achieved by local administration (inhalation therapy). *In vivo,* the sizes of the biofilms are smaller than in most of the *in vitro* models (Høiby et al., 2015) and the local biofilm environments may be quite variable, depending on the site of infection. Therefore, different antibiotic tolerance mechanisms may be the underlying cause of the survival of bacteria in different types of biofilm infections. For example, in cystic fibrosis patients, the *P. aeruginosa* biofilms are not surface-attached but present as clusters of cells in the airway mucus (Bjarnsholt et al., 2013), and surrounded by inflammatory polymorphonuclear cells, which are consuming a large proportion of the available oxygen (Kolpen et al., 2010; Jensen et al., 2017). Accordingly, evidence has been provided that the bacteria in these biofilms experience anaerobic conditions (Worlitzsch et al., 2002; Aanaes et al., 2011). On the contrary, the *P. aeruginosa* biofilms which are attached to endotracheal tubes causing nosocomial ventilator-associated pneumonia in patients in intensive care units are most likely localized in an aerobic environment, suggesting that the bacteria in the periphery of these biofilms experience aerobic conditions (Høiby et al., 2015).

*In vivo*, the complexity of the tolerance of biofilms to antibiotics might be increased by the heterogeneity of the biofilm-forming clinical strains with different gene expression patterns due to clonal evolution and adaptation during chronic infections such as in the chronic lung infection with *P. aeruginosa* in CF patients (Rossi et al., 2018).

Multispecies biofilms are frequently encountered in nature but their significance in chronic infections is unclear as imaging of *in vivo* biofilms in many cases showed monospecies bacterial aggregates, except for oral and intestinal biofilms (Burmolle et al., 2010). It has been reported that *in vitro* established multispecies biofilms, including *P. aeruginosa,* displayed increased tolerance to antibiotics compared to monospecies biofilms (Ryan et al., 2008; Tavernier et al., 2017).

## Tolerance of Biofilms to Different Antibiotic Classes

In this section, we describe biofilm-associated antimicrobial tolerance mechanisms that play a role in tolerance to specific antibiotic classes. This emphasizes the multifaceted nature of biofilm-associated antibiotic tolerance.

### Biofilm-Associated Tolerance Toward Beta-Lactam Antibiotics

The *in vitro* testing of minimal biofilm inhibitory concentration of beta-lactams showed up to 1,000-fold higher concentrations compared to planktonic MIC (Moskowitz et al., 2004; Macia et al., 2014). In general, beta-lactams have poor anti-biofilm effect. This is due to the beta-lactams mode of action targeting the peptidoglycan synthesis and being effective only on actively growing, dividing cells. Thus, the primary tolerance mechanism of biofilms to beta-lactams is related to the slow growth of bacteria in biofilms (Figure 1).

The induction of beta-lactamase transcription or surplus beta-lactamase released from killed bacteria in the outer susceptible biofilm layer in response to the presence of a beta-lactam antibiotic impairs the penetration of the beta-lactam molecules through the biofilm layers (Dibdin et al., 1996; Bagge et al., 2004a). Using a translational fusion of *ampC* with an unstable green fluorescent protein reporter, Bagge et al. (2004a) demonstrated that in the absence of beta-lactam antibiotic, the level of *ampC*-encoded beta-lactamase in *P. aeruginosa* biofilms is negligible. However, expression of the enzyme was found in the periphery of the biofilm when induced with low concentrations of imipenem (a strong inducer of *ampC*), while high concentrations of imipenem led to induction of *ampC* throughout the biofilm. The inactivation of the beta-lactam molecules by the enzymes in the biofilm matrix has pharmacokinetic/pharmacodynamic consequences, as the time-dependent effect of ceftazidime, a cephalosporin that is hydrolyzed by the AmpC beta-lactamase of *P. aeruginosa*, was changed to a dose-dependent effect (Hengzhuang et al., 2013), suggesting that beta-lactams have to be used in high dosages in order to overwhelm the degradative capacity of the enzymes (Bowler et al., 2012). Alternatively, treatment of biofilm infections with combination of beta-lactams and beta-lactamase inhibitors (such as ceftolozane/tazobactam or ceftazidime/avibactam (Torrens et al., 2016) or beta-lactamase stable compounds, like the carbapenem meropenem, can overcome this tolerance mechanism. In accordance, meropenem showed good *in vitro* activity on biofilms of *P. aeruginosa* (Moskowitz et al., 2004).

### Biofilm-Associated Tolerance Toward Fluoroquinolone Antibiotics

*In vitro*, fluoroquinolones are much more active against biofilm bacteria than beta-lactams and the minimal biofilm inhibitory concentrations (MBIC) of *P. aeruginosa* are more similar to the planktonic MICs (Moskowitz et al., 2004; Macia et al., 2014) than for other antibiotics. As fluoroquinolones have good anti-biofilm effect as well as good tissue penetration, and are available for oral administration, they are frequently used in combination therapy for treatment of biofilm infections such as bone and joint implant-associated infection and chronic lung infections (Høiby et al., 2015; Ciofu et al., 2017).

Quinolones are uncharged molecules and diffuse easily through the biofilm matrix. *In vitro* study of quinolones on sub-optimally growing or non-growing *P. aeruginosa* showed significant bactericidal activity (Eng et al., 1991). However, the low oxygen concentration in biofilms affects the bactericidal effect of quinolones, probably due to formation of ROS in insufficient levels to cause bactericidal effect (Figure 1). The low oxygen level seems to be the primary mechanism for the tolerance of biofilms to quinolones together with adaptive responses such as SOS and the stringent response (Stewart et al., 2015). It has been shown that targeting the low oxygen tension by exposure of biofilms to hyperbaric oxygen therapy (HBOT) improves the anti-biofilm effect of quinolones (Kolpen et al., 2017; Gade et al., 2018).

### Biofilm-Associated Tolerance Toward Aminoglycoside Antibiotics

A plethora of mechanisms appear to play a role in the tolerance of *P. aeruginosa* biofilms toward aminoglycoside antibiotics (Figure 1). These mechanisms include binding of the aminoglycosides by various components of the biofilm matrix, as well as expression of specific genes that confer biofilm-associated aminoglycoside tolerance.

Alginate may act as a shield against aminoglycoside antibiotics in biofilms formed by alginate over-producing *P. aeruginosa* strains. Thus, evidence was provided that biofilms formed by an alginate over-producing *P. aeruginosa* strain were significantly more tolerant to tobramycin than biofilms formed by the isogenic non-mucoid strain (Hentzer et al., 2001). Furthermore, it has been shown that the Psl exopolysaccharide affords some degree of biofilm-associated tolerance toward aminoglycosides. In one study, the protective effect of Psl against tobramycin was pronounced in young *P. aeruginosa* biofilms (Billings et al., 2013), but other studies have provided evidence for a role of Psl in tobramycin tolerance of mature *P. aeruginosa* biofilms as well (Yang et al., 2011). The Pel exopolysaccharide can also provide protection against aminoglycosides in *P. aeruginosa* biofilms. Thus, biofilms formed by mutants lacking Pel were found to be more susceptible to tobramycin and gentamycin than the corresponding wild-type biofilms (Colvin et al., 2011). Moreover, eDNA can also play a role in hindering penetration of aminoglycosides into *P. aeruginosa* biofilms. DNA can bind positively charged antibiotics such as aminoglycosides (Ramphal et al., 1988; Hunt et al., 1995), and accordingly Chiang et al. (2013) provided evidence that biofilms formed by a DNA-release deficient *P. aeruginosa* quorum-sensing mutant were susceptible to tobramycin, but became tobramycin tolerant if they were supplied with DNA that incorporated into the biofilm.

Aminoglycoside tolerance of *P. aeruginosa* biofilms has also been linked to extracellular DNA-mediated activation of the *pmr* and *arn* genes through binding of magnesium as well as local acidification (Mulcahy et al., 2008). The *arn* gene products confer aminoglycoside tolerance through the addition of aminoarabinose to the lipid A moiety of LPS (Lewenza, 2013). In addition, the PmrA-regulated PA4773-4775 locus codes for enzymes that catalyze synthesis of spermidine, which is a polyamine that localizes to the outer membrane and reduce outer membrane permeability for aminoglycosides (Johnson et al., 2012).

The *ndvB* gene was identified to be involved in tolerance of *P. aeruginosa* PA14 biofilms toward tobramycin and gentamicin through a transposon mutant screen (Mah et al., 2003). An *ndvB* mutant was less tolerant to tobramycin and gentamicin than the wild type when grown in biofilms, but when grown planktonically the *ndvB* mutant and wild type were equally susceptible to the aminoglycosides. The mechanism appeared to be biofilm-specific due to specific expression of the *ndvB* gene in biofilm cells compared to planktonic cells (Mah et al., 2003; Beaudoin et al., 2012). Tolerance mediated by *ndvB* was shown to be due to drug sequestration by cyclic periplasmic glucans, as well as due to a role in the activation of ethanol oxidation genes (Mah et al., 2003; Beaudoin et al., 2012).

A number of the previously described mechanisms that involve expression in biofilms of specific antibiotic tolerance-mediating genes like *brlR* and PA1874-1877 confer tolerance to aminoglycosides (Mah et al., 2003; Zhang and Mah, 2008; Liao and Sauer, 2012; Poudyal and Sauer, 2018a). In addition, as described previously, the stringent response in *P. aeruginosa* has been suggested to play a role in the tolerance of biofilms to various antibiotics, including aminoglycosides, by reducing oxidative stress (Nguyen et al., 2011).

The lack of oxygen and low metabolic rate of the bacterial cells in the deeper layers of the biofilms also plays a role in the tolerance of biofilms to aminoglycosides, as aminoglycosides require an active, oxygen-dependent up-take through the cytoplasmic membrane in order to reach their ribosomal target, and lack of oxygen impair the membrane potential and the transport of these molecules into the cytoplasm (Figure 1; Verklin and Mandell, 1977; Taber et al., 1987; Stewart et al., 2015). Accordingly, that targeting the low oxygen tension by exposure of biofilms to hyperbaric oxygen therapy (HBOT) improves the anti-biofilm effect of aminoglycosides both *in vitro* (Møller et al., 2019) and *in vivo* (Lerche et al., 2017). In addition, it has also been proposed that a set of metabolites, such as fumarate, which promote TCA cycle activity, could potentiate tobramycin lethality in *P. aeruginosa* bacteria with low-metabolic activity (persisters) (Koeva et al., 2017).

### Biofilm-Associated Tolerance Toward Antimicrobial Peptides

Most antimicrobial peptides are positively charged similar to the aminoglycosides. Therefore, some of the mechanisms that are involved in the tolerance of *P. aeruginosa* biofilms toward aminoglycosides may also play a role in biofilm tolerance to antimicrobial peptides. For example, it may be expected that binding of antimicrobial peptides by the negatively charged biofilm matrix components, such as eDNA, can play a role in tolerance of *P. aeruginosa* biofilms toward antimicrobial peptides. In addition, the Psl exopolysaccharide has been shown to confer protection of *P. aeruginosa* biofilms against colistin and polymyxin B (Billings et al., 2013). Moreover, evidence has been provided that the stringent response plays a role in tolerance of *P. aeruginosa* biofilms toward colistin (Nguyen et al., 2011; Khakimova et al., 2013).

As described previously, upregulation of the *pmr* and *arn* genes in *P. aeruginosa* biofilms may occur through eDNA-mediated cation chelation and acidification (Mulcahy et al., 2008; Wilton et al., 2016). Moreover, evidence has been provided that exposure of *P. aeruginosa* biofilms to colistin leads to upregulation of the *pmr* and *arn* genes in the metabolically active peripheral subpopulation (Pamp et al., 2008). The *arn* gene products mediate a reduction of the net negative charge of LPS, which reduces the interaction with and uptake of polymyxins (Lewenza, 2013). Upregulation of the *pmr/arn* operon plays a role in colistin tolerance of *P. aeruginosa* biofilms together with activation of MexAB-OprM efflux pump (Pamp et al., 2008). In addition, Chiang et al. showed that also the efflux pumps encoded by the *mexCD-oprJ* and *muxABC-opmB* genes are expressed in response to colistin exposure (Figure 1; Chiang et al., 2012). Colistin could not eradicate the metabolically active peripheral subpopulation, but was efficient in eliminating the metabolically inactive subpopulation in the deeper layers of the biofilm (Figure 2C; Pamp et al., 2008). This is in agreement with data showing the increased bactericidal activity of colistin on *P. aeruginosa* biofilms grown under anaerobic conditions (Kolpen et al., 2016).

The combination of colistin targeting the metabolically inactive population with antibiotics targeting the metabolically active population, such as ciprofloxacin and tobramycin, has been shown to be able to eradicate *P. aeruginosa* biofilms *in vitro* (Figure 2; Pamp et al., 2008) and *in vivo* (Herrmann et al., 2010). In cystic fibrosis patients, eradication therapy with colistin or tobramycin inhalations and oral ciprofloxacin are used to treat *P. aeruginosa* lung infections after the first identification of the bacteria in airway secretions (Høiby et al., 2015).

It has been shown that sub-MIC concentrations of the different classes of antibiotics can stimulate biofilm formation both in Gram positive and Gram negative bacteria. The involved mechanisms have been reviewed recently (Ranieri et al., 2018) and include eDNA release, induction of phage elements and a variety of regulatory responses. For example in the case of *P. aeruginosa* imipenem can induce production of alginate (Bagge et al., 2004b), ciprofloxacin can induce SOS response and tobramycin can induce biofilm formation through c-di-GMP with increased tolerance of the biofilm (Hoffman et al., 2005).

## Development of Antibiotic Resistance in Biofilms

The described tolerance mechanisms, all contribute to the persistence of biofilms, which provide a fertile ground for the emergence of antibiotic-resistant mutants. In planktonic cultures, it has been reported that tolerance precedes the occurrence of resistance (Levin-Reisman et al., 2017).

Antibiotic resistance implies mutations in resistance genetic determinants leading to increased antibiotic minimal inhibitory concentrations for bacterial cells disrupted from biofilm and it is accepted as a side-effect of prolonged maintenance antibiotic therapy.

In contrast to the planktonic, fast-dividing cells that are traditionally used to study antibiotic resistance development in shaking cultures, biofilm-grown bacteria encounter gradients of nutrients and oxygen which lead to a heterogeneous bacterial population including slow-growing or non-dividing cells (Stewart, 2015; Stewart et al., 2016). Knowledge of mutagenesis in non-dividing or nutrient-deprived cells (stationary-phase or adaptive mutagenesis (Foster, 2007; Sekowska et al., 2016)) suggests that the mutation rate will be higher and represented by different types of mutations in biofilms compared to planktonic cultures (Bjedov et al., 2003; Bharatan et al., 2004; Kivisaar, 2010; Maharjan and Ferenci, 2017). The mechanisms responsible for the increased biofilm mutagenesis involve oxidative stress (Boles and Singh, 2008), SOS-responses to DNA-damage and RpoS-dependent responses (Bjedov et al., 2003; Kivisaar, 2010). Compared to planktonic, homogenous populations, the spatially structured and heterogeneous environment of the biofilms can be considered as a collection of different niches with different selective pressures (Rainey et al., 2000; Kassen and Rainey, 2004; Melnyk et al., 2015) which provide the opportunity of a larger variety of resistant mutants to persist, without competition in the biofilm (Perron et al., 2007).

A common trait observed in biofilm evolution experiments is the diversification of the bacterial population to a higher degree than encountered in evolution studies using planktonic cultures (Steenackers et al., 2016). Another characteristic of the evolution in biofilms, especially observed during chronic infections, is the frequent isolation of bacteria with high mutation rates, e.g. hypermutators due to mutations in their DNA-repair mechanisms (Oliver et al., 2000; Labat et al., 2005; Macia et al., 2005; Daurel et al., 2007). This occurs in spite of the generally accepted fact that mutator phenotypes are self-limiting due to the high risk of deleterious mutations (Denamur and Matic, 2006). This suggests that mutators are maintained in some biofilm niches and are probably compensating for the slow bacterial growth by ensuring a pool of spontaneous mutants that allow adaptation of the biofilm and emergence of resistance in biofilms. It has previously been shown that when adaptation is limited by the supply of mutations, selection favors strains with increased mutation rates owing to mutational inactivation of the mismatch repair system (Bjedov et al., 2003). An enhanced adaptability of the mutators over the wild type was observed when grown in competition experiments in flow-cell biofilms (Lujan et al., 2011; Macia et al., 2011).

**Figure 3:**
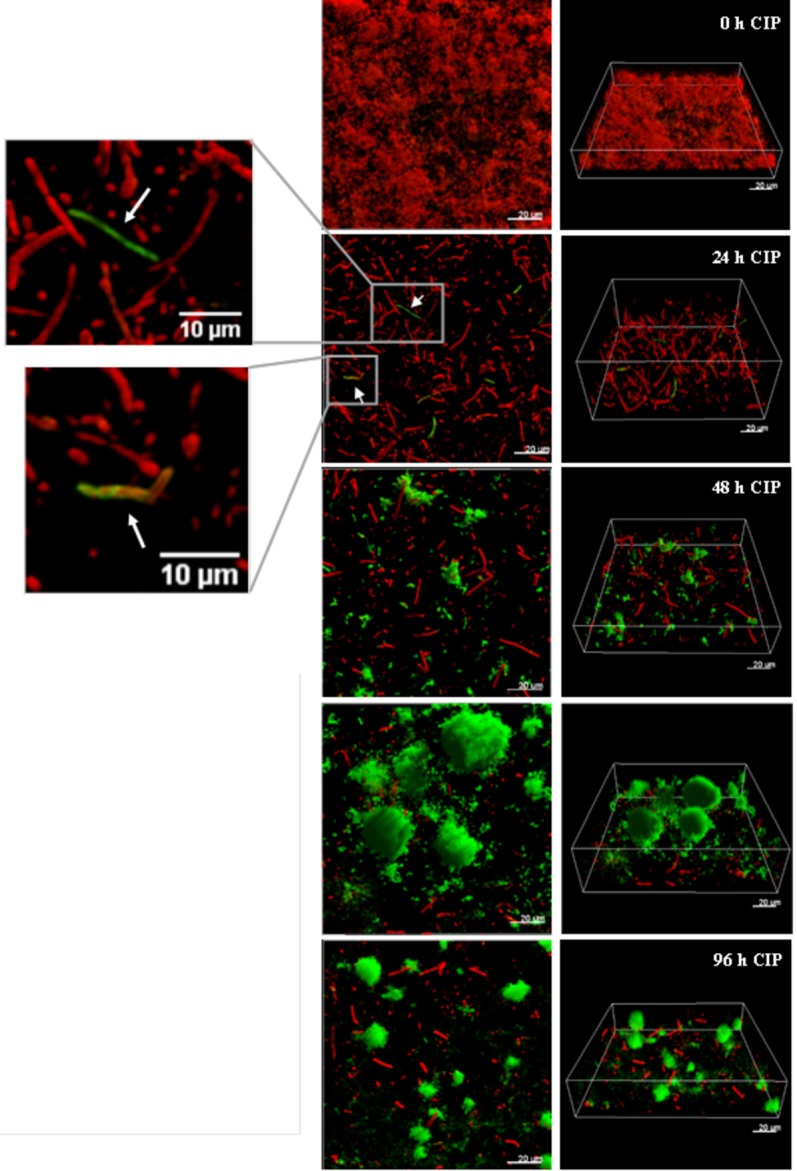
The development of *nfxB* mutants in 72 h-old PAO1 flow-cell biofilm during treatment with low-dose ciprofloxacin. Biofilms of PAO1 *mCherry*-P_CD_-*gfp* (*mCherry* integrated at the *attB* site; mini-Tn7::P_CD_-*gfp* inserted upstream of the *glmS* gene) were grown in three independent channels of flow-cell chambers for 72 h and were then treated with 0.2 μg/ml CIP for a total of 96 h. Imaging by CLSM was done every 24 h. Red color represents wild-type cells due to constitutive expression of *mCherry*, whereas green color shows *nfxB* mutants due to expression of the P_CD_-*gfp* reporter which is upregulated specifically in these mutants. The images show orthogonal 3D biofilm views (left panel) or perspective view (right panel) with overlay of red and green channel fluorescence (Zaborskyte et al., 2016). Figure is reproduced from Antimicrobial Agents and Chemotherapy, 61, Issue 3, e02292–16 with permission from the American Society of Microbiology.

It was recently shown by *in vitro* experimental evolution studies that antibiotic resistance developed by distinct pathways in planktonic cultures and biofilms exposed to sub-inhibitory levels of ciprofloxacin (Ahmed et al., 2018). While the size of the ciprofloxacin resistant subpopulation was higher in biofilms, the levels of resistance (MICs) were lower compared to planktonically evolved cultures, which correlate to mutations in efflux pumps in biofilms and mutations in target genes in planktonic cultures. A rapid emergence of *nfxB* mutants overexpressing the MexCD-OprJ efflux pump in biofilms exposed to low doses of ciprofloxacin, with mutants comprising 80–90% of the biofilm population after 4 days treatment, was also demonstrated (Figure 3; Zaborskyte et al., 2016). This suggests that biofilm growth promotes the occurrence and maintenance of low-level resistance mutants, representing the first-step mutations with potential accumulation of mutations leading to increased MIC at later steps.

## Conclusions and Perspectives

The ESKAPE pathogen *Pseudomonas aeruginosa,* is an important cause of persistent infections due to its ability to form biofilms which display tolerance and resistance to antimicrobial agents. The multiple and various mechanisms employed by *P. aeruginosa* to survive antibiotic treatment while growing in a biofilm represent an important therapeutic challenge. The molecular mechanisms underlying tolerance of biofilms to antibiotics are targets for therapeutic interventions for potentiating the anti-biofilm effect of antibiotics. To identify the best intervention targets, a clarification of the relative contribution of the mechanisms involved in the tolerance of biofilms to specific classes of antibiotics is needed. In addition, different tolerance mechanisms might be triggered *in vivo* at the biofilm infection site depending on the specific environmental conditions, such as the access to nutrients and oxygen, which depend on several factors such as tissue vascularization, and degree and type of inflammation (Jensen et al., 2017). Thorough understanding of the relative importance of the antibiotic tolerance mechanisms for the persistence of different types of biofilm infections requires further research. Knowledge of the complexity of biofilm-specific antibiotic tolerance and of the biofilm-specific dynamics of antibiotic resistance evolution will ultimately provide a basis for the development of therapeutic solutions for patients suffering from chronic biofilm infections.

## Author Contributions

OC and TT-N contributed equaly to the concept and writing of the review.

### Conflict of Interest Statement

The authors declare that the research was conducted in the absence of any commercial or financial relationships that could be construed as a potential conflict of interest.
